# Remarkable Disease of the Heart

**Published:** 1831-01-01

**Authors:** 


### XXIX.

### Remarkable Disease of the Heart. By Dr. Buet.

A man, 45 years of age, of delicate constitution, narrow chest, and subject to palpitations for many years, with dyspnœa on walking quick or going up an ascent. To these were added habitual cough, sometimes dry, sometimes with expectoration. The digestive organs were often disordered, and there was considerable emaciation. The pulse was irregular, and auscultation aided by percussion shewed the heart greatly enlarged, the pulsations being loud and tumultuous, with a confused noise of bruit de soufflet and bruit de scie. In front, the respiration was entirely masked by the soundsof the heart, excepting under the left clavicle, and in the right side of the thorax, where it was sufficiently audible throughout. In the left side respiration only audible at the top of the lung. The *right* sounded well, except at one place near the nipple. The sound of the *left* side was dull pretty generally, except under the clavicle. The diagnosis formed was, great dilatation of the right ventricle, state of its parietes uncertain — dilatation with hypertrophy of the left ventricle —probable narrowing of the auriculo-ventricular opening of this side—the cardiac disease the principal one—lower half of left lung engorged—upper portion, as well as the whole of the right lung respirable. The case was, of course, pronounced to be fatal, and only mitigation to be attempted. Leeches were occasionly applied to the præcordial region, and digitalis with diluents were ordered internally. After some days the patient found himself greatly relieved—the dyspnœa disappeared — the irregularities of the pulse ceased, and never afterwards returned — the cough abated — the expectoration decreased. This was toward the close of last September. By the middle of December, he was free from cough and expectoration, and the dyspnœa was inconsiderable. The impulsion of the heart was also much diminished—appetite good, and the patient congratulated himself on the improving state of his health. Nevertheless the emaciation went on, and his strength became reduced. After this, the old symptoms gradually recommenced, and by the beginning of March last, he was forced to keep his bed. The abdomen swelled a little, as did the ankles. The remainder of the scene may be easily imagined.

*Dissection.* There was no serous effusion in any of the cavities, although the members were greatly infiltrated. Here M. Buet lauds the pleximeter of M. Piorry, and declares that by it the slightest degree of effusion in the thorax may be detected. M. Piorry examined the dead body before the chest was opened, and pronounced the absence of all effusion. This was verified on examination. The heart was enormously enlarged—at least equal to the size of a child's head. There was a large quantity of black-blood. The right auricle and ventricle were greatly dilated, double, at least, the usual size, while their parietes were very thin, that of the auricle not thicker than paper. The *left* ventricle was dilated to double the size of the right one, while its parietes were hypertrophied to the extent of an inch in thickness—of the densest structure, and deepest colour. The muscular fibres of this side of the organ were remarkably developed, and offered great resistance to the scalpel. The left auricle was in its natural state. The aorta was neither dilated nor contracted. The lung of the right side was slightly engorged but crepitant throughout. The upper half of the *left* lung resembled that of the right; but all the rest was hepatized in the last degree. There were neither tubercles nor suppuration. No effusion into the abdomen.

Was this a case where Baron Larrey's mode of treatment was applicable? Whatever influence the counter-irritation of the Baron might have had on the hypertrophy of the left side of the heart, it could not have been of any advantage to the passive dilatation of the right chambers. The Baron would, however, have put his favourite measures in force, but with what success we will not pretend to divine.

